# Effects of Semantic Richness on Lexical Processing in Monolinguals and Bilinguals

**DOI:** 10.3389/fnhum.2016.00382

**Published:** 2016-07-29

**Authors:** Vanessa Taler, Rocío López Zunini, Shanna Kousaie

**Affiliations:** ^1^School of Psychology, University of Ottawa, OttawaON, Canada; ^2^Bruyère Research Institute, OttawaON, Canada

**Keywords:** semantic richness, number of senses, polysemy, bilingualism, event-related potential

## Abstract

The effect of number of senses (NoS), a measure of semantic richness, was examined in monolingual English speakers (*n* = 17) and bilingual speakers of English and French (*n* = 18). Participants completed lexical decision tasks while EEG was recorded: monolinguals completed the task in English only, and bilinguals completed two lexical decision tasks, one in English and one in French. Effects of NoS were observed in both participant groups, with shorter response times and reduced N400 amplitudes to high relative to low NoS items. These effects were stronger in monolinguals than in bilinguals. Moreover, we found dissociations across languages in bilinguals, with stronger behavioral NoS effects in English and stronger event-related potential (ERP) NoS effects in French. This finding suggests that different aspects of linguistic performance may be stronger in each of a bilingual’s two languages.

## Introduction

It is now well-established that lexical processing is influenced by semantic richness or the amount of semantic information a word possesses. A word’s semantic richness may be measured in multiple ways, including imageability, number of features, number of senses, number of associates (NoA), semantic neighborhood density (or number of semantic neighbors), and body–object interaction. A growing body of research has indicated that semantic richness facilitates lexical processing: semantically rich words are recognized more quickly and accurately than words that are more semantically impoverished (e.g., Buchanan et al., 2001; Duñabeitia et al., 2008; Pexman et al., 2008; Yap et al., 2011). These effects are observed across multiple dimensions and tasks (Yap et al., 2011, 2012), indicating that semantic richness is a robust predictor of word processing.

Several studies have also shown processing differences between semantically rich and impoverished words at the neurophysiological level (Kounios et al., 2009; Muller et al., 2010; Amsel, 2011; Laszlo and Federmeier, 2012; Rabovsky et al., 2012; Amoruso et al., 2013; Amsel and Cree, 2013). These studies used event-related potentials (ERPs), a technique with temporal resolution on the order of milliseconds, which is ideal for studying online language processing. These studies have focused primarily on the N400, a negative-going ERP component peaking at approximately 400 ms post-stimulus onset that is consistently modulated by semantic processing (Kutas and Federmeier, 2011).

Kounios et al. (2009) and Amsel and Cree (2013) found larger N400 amplitudes for words with a low number of features than for words with a high number of features, suggesting greater ease of processing of words with a high number of features. However, other studies investigating number of features have found the opposite effect (Amsel, 2011; Rabovsky et al., 2012). ERP studies that investigated the variable NoA have found larger N400 amplitudes for words with high than low NoA (Muller et al., 2010; Laszlo and Federmeier, 2012). The discrepancy in findings may be due to a number of factors including type of language task (lexical decision vs. semantic categorization), type of analyses (general linear model statistics vs. mixed-effects models), and the semantic richness variable under study (e.g., the effects of number of features may differ from the effects of NoA because they measure different aspects of the semantic information associated with a word).

To our knowledge, only one ERP study has investigated number of senses (Taler et al., 2013). Consistent with previous behavioral research, findings revealed faster reaction times for words with high than low number of senses during a lexical decision task in monolingual young adults. In addition, words with many senses exhibited smaller N400 amplitudes relative to words with few senses.

The majority of research into the effects of semantic richness on lexical processing has focused either on monolingual speakers or has not specified the language background of study participants. A large body of research, however, indicates differences in language function between monolinguals and bilinguals. Monolinguals consistently outperform bilinguals in language tasks (for a review, see Bialystok, 2009). Semantic organization has also been shown to differ between monolinguals and bilinguals. Antón-Méndez and Gollan (2010) found differences in associative effects between bilinguals and monolinguals: in a word association task, bilinguals’ responses were less typical than those of monolinguals (for example, in response to the target “bride,” bilinguals produced the item “dress” while monolinguals were more likely to produce “groom”). Bilinguals’ performance was similar to that of monolinguals when the associate was high frequency but not when the associate was low frequency. That is, bilinguals were more affected by frequency information than monolinguals. Similarly, Johns et al. (2016) demonstrate that semantic diversity information—that is, the number of contexts in which a word occurs—exerts a stronger effect on lexical decision performance in bilinguals than monolinguals.

In the current study, we aimed to extend previous research on semantic richness effects to a bilingual population, an area that remains unexplored in the literature. We used behavioral and ERP measures to examine sense relatedness effects in highly proficient bilingual speakers of English and French, and compare effects to those we previously reported for English-speaking monolinguals (Taler et al., 2013). Bilinguals completed testing in both English and French, allowing us to compare semantic richness effects in each language, as well as to compare bilinguals’ performance in first and second languages (L1 and L2). Because bilinguals have reduced exposure to each language, it is likely that the semantic knowledge possessed by bilingual speakers is more impoverished in each of their languages relative to monolingual speakers, as has been shown in children (Cremer and Schoonen, 2013). We thus hypothesized that semantic richness effects would be weaker in bilingual than monolingual speakers. We also predicted stronger effects of semantic richness in bilinguals’ L1 than in their L2.

## Materials and Methods

### Participants

Participants were 17 right-handed native monolingual English speakers^1^ (nine females) and 18 right handed English-French bilinguals (10 females). Participants were recruited through word of mouth and advertising on the campus of the University of Ottawa. All participants were undergraduate students at the University of Ottawa. Monolinguals and bilinguals were matched on age (*p* = 0.16) and education (*p* = 0.39). All participants completed a self-report health and history questionnaire to ensure that they were in good health and not taking any medication that could affect cognitive function. Participants also completed a short language battery to ensure normal language performance and high proficiency in both languages in the bilingual participants. See **Table 1** for complete demographic and language performance information. The research protocol was approved by the Institutional Review Boards of Bruyère Research Institute and the University of Ottawa and was conducted according to the principles expressed in the Declaration of Helsinki (protocol number M16-11-007). All participants provided written informed consent.

**Table 1:** Demographic and language performance information in monolinguals and bilinguals.

Group	Monolingual (*n* = 17; nine females) [Mean (*SD*)]	Bilingual (*n* = 18; 10 females) [Mean (*SD*)]	Monolinguals vs. Bilinguals^a,b^ (*p*-value)	Bilingual L1 vs. L2^c^ (*p*-value)
Age (years)	22.06 (*2.1*)	21.11 (*1.71*)	0.16	
Education (years)	15.71 (*1.0*)	15.33 (*1.46*)	0.39	
L1		10 English 8 French		
Self-ranking (L, R, S, and W)^d^	5 (*0*), 5 (*0*), 5 (*0*), 5 (*0*)	L1: 5 (*0*), 4.9 (*0.24*), 5 (*0*), 4.8 (*0.43*)		0.09, 0.09,
		L2: 4.6 (*0.97*), 4.5 (*1.0*), 4.3 (*1.1*), 4.3 (*1.1*)		0.02, 0.04
Boston Naming Test	53.69 (*3.23*)	L1: 47.76 (*6.9*)	<0.01	45
		L2: 44.47 (*8.9*)		
Category fluency (Animal)	26.63 (*5.85*)	L1: 23.73 (*6.31*)	0.20	0.62
		L2: 22.38 (*5.83*)		
Letter fluency (A, F, S)	44.13 (*14.70*)	L1: 35.75 (*8.25*)	0.06	0.98
		L2: 34.20 (*8.73*)		


*^a^Monolinguals compared to bilinguals in their L1 for language tasks. ^b^Independent samples *t*-test. ^c^Paired samples *t*-test. ^d^L, listening; S, speaking; R, reading; W, writing; self-rated on a Likert scale where 1 = no ability at all and 5 = native-like ability.*

### Materials

#### Language Battery

Participants completed a short language battery that consisted of self-reported language proficiency ratings, the Boston Naming Test (BNT; Kaplan et al., 1983), and two verbal fluency measures (letter and category).

##### Self-reported language proficiency

Participants were asked to rate their proficiency on different aspects of their language using a 5-point Likert scale where 1 indicated “no ability at all” and 5 indicated “native-like ability.” Monolinguals rated their native language, and bilinguals rated both of their languages.

##### BNT

The BNT is a picture naming tasks that requires participants to consecutively name 60 line drawings that increase in difficulty as the task progresses. Monolinguals performed this task in English only, while bilinguals completed it in both English and French.

##### Verbal Fluency

The verbal fluency tasks required participants to generate as many exemplars at they could for the given letter (letter fluency: letter F, A, and S) or category (category fluency: animals). Monolinguals performed this task in English only, and bilinguals performed it in English and in French.

#### Experimental Tasks

The experimental tasks were two lexical decision tasks: one in English (performed by monolinguals and bilinguals) and one in French (performed by bilinguals only). In each task, stimuli were presented in 18-point Courier New font on a black background. Each trial started with a fixation cross presented at the center of the screen for 500 ms followed by a target stimulus presented for 2000 ms or until the participant made a response. Stimuli were presented using E-prime 2.0 software (Psychology Software Tools, Pittsburgh, PA, USA) on a Dell Optiplex 780 desktop computer with Windows XP Professional operating system.

In each task, there were 140 stimuli, 70 real words and 70 pseudowords. Half of the real words had many related senses (high condition) and the other half had few related senses (low condition). For the English task, number of senses (NoS) was determined using WordNet (Princeton University, 2010). High NoS and low NoS words were matched for length, familiarity and imageability from the MRC Psycholinguistic Database (Coltheart, 1981), and subtitle frequency and orthographic neighborhood density from the English Lexicon Project database (Balota et al., 2002). English pseudowords were matched to English words for length and orthographic neighborhood density. See **Table 2** for English stimulus characteristics. NoS for French stimuli was determined using a dictionary search; English and French stimuli were matched for NoS, and French high and low NoS were matched for length and subtitle frequency using norms from Lexique (New et al., 2001), and subjective frequency, imageability, and orthographic neighborhood density using norms from Omnilex (Desrochers, 2006). French pseudowords were phonotactically legal and matched to real words for length. See **Table 3** for French stimulus characteristics.

**Table 2:** Psycholinguistic properties of English items.

	High number of senses	Low number of senses	Pseudowords
Subtitle frequency	53.95 (65.41)	27.24 (47.02)	n/a
Length	5.17 (1.36)	5.55 (1.33)	5.51 (1.34)
Familiarity	541.66 (48.90)	532.39 (46.18)	n/a
Imageability	570.40 (50.49)	592.97 (46.19)	n/a
Number of senses	6.83 (2.53)	1.70 (0.47)	n/a
Orthographic neighborhood density	4.80 (4.44)	3.20 (3.93)	3.69 (3.63)


*Subtitle frequency and orthographic neighborhood density taken from the English Lexicon Project (Balota et al., 2002). Familiarity and imageability norms taken from the MRC Psycholinguistic Database (Coltheart, 1981). Total number of items for which a score was available.*

**Table 3:** Psycholinguistic properties of French items.

	High number of senses	Low number of senses	Pseudowords
Subtitle frequency	35.36 (15.01)	71.33 (28.84)	n/a
Length	6.24 (6.74)	6.73 (1.64)	7.30 (1.27)
Subjective frequency	4.40 (1.57)	4.16 (1.79)	n/a
Imageability	4.56 (1.77)	4.84 (1.89)	n/a
Number of senses	6.77 (1.63)	1.63 (0.49)	n/a
Orthographic neighborhood density	3.41 (3.68)	2.12 (3.29)	n/a


*Norms were taken from Omnilex (Desrochers, 2006).*

### EEG Recording

The EEG was recorded from 32 tin electrodes positioned according to the international 10–20 system of electrode placement using a commercially available nylon cap (Electro-Cap International, Inc., Eaton, OH, USA). A cephalic site was used as the ground and the electrodes were referenced online to linked ears. Two electrodes were placed at the outer canthus of each eye to record horizontal electro-oculogram (EOG) activity, and two additional electrodes were placed above and below the left eye to record vertical EOG activity. The EEG was amplified with NeuroScan NuAmps (Neuroscan, El Paso, TX, USA) and was sampled at a rate of 500 Hz in a DC to 100 Hz bandwidth. Impedances were kept below 5 kΩ. The EEG data were processed using Neuroscan 4.3 EDIT software (Neuroscan, El Paso, TX, USA). A 30 Hz low pass filter was applied, vertical EOG artifacts were corrected using a spatial filter (NeuroScan EDIT 4.3), trials containing horizontal EOG deflections exceeding ±50 μV, and trials with deflections exceeding ±100 μV at the electrode sites of interest were excluded from averages. Epochs were 1100 ms long with a 100 ms pre-stimulus baseline. Averages were computed based on the experimental condition NoS (high, low) for both English and French tasks and were baseline corrected to 0 μV average of the 100 ms pre-stimulus interval. Only correct trials were included in analyses.

### Procedure

Participants were tested in two sessions. In session one, participants performed the language battery to ensure normal language function and high second language proficiency in the bilinguals. In session two, monolinguals and bilinguals performed an English lexical decision task, and bilinguals performed an additional French lexical decision task while EEG was recorded. The order of French and English tasks was counterbalanced. The experimental tasks took approximately 5 min each to complete and were followed by an additional task that was part of a separate study not reported here and that took approximately 30 min to complete. Participants were compensated $10 per hour of participation.

## Results

Results were analyzed as follows: first, we ran a series of analyses comparing monolinguals to bilinguals in the English experiment. We then compared monolinguals to bilinguals in their first language (L1). Bilinguals’ performance in the English and French experiments was then compared. A final series of analyses was conducted comparing bilinguals in their L1 and L2. All analyses were conducted using SPSS Version 22. Results of each analysis are provided below.

### Behavioral Results

Mixed ANOVAs were performed for group comparisons (monolinguals vs. bilinguals) while repeated measures ANOVAs were performed for language comparisons within the bilingual group. Significant interactions were decomposed with Bonferroni corrected simple effects comparisons. Analyses are described in detail in the sub-sections below.

#### Monolinguals vs. Bilinguals: Performance in English

Reaction time (RT) and accuracy were analyzed with two separated mixed ANOVAs with the within-subject factor Number of Senses (NoS: high vs. low) and the between-subject factor Group (monolingual vs. bilingual). Reaction time analyses revealed a main effect of NoS [*F*(1,33) = 20.25, *MSE* = 552.25, *p* = 0.01, $\eta_{p}^{2}$ = 0.38], indicating faster reaction times for high than low NoS words. The main effect of Group was not significant (*F* < 1). However, there was a trend toward an interaction between Group and NoS [*F*(1,33) = 3.81, *MSE* = 552.25, *p* = 0.06, $\eta_{p}^{2}$ = 0.10]. Planned simple effects comparisons revealed that the RTs were faster for words with high NoS relative to low NoS in the monolingual group only.

Accuracy analyses revealed a main effect of NoS [*F*(1,33) = 9.41, *MSE* = 5.25, *p* < 0.01, $\eta_{p}^{2}$ = 0.22], demonstrating more accurate responses for higher than low NoS items. There was also an interaction between Group and NoS [*F*(1,33) = 4.60, *MSE* = 5.25, *p* = 0.04, $\eta_{p}^{2}$ = 0.12], indicating that bilinguals were less accurate for words with low NoS – that is, bilinguals responded more accurately to high than low NoS words (*p* < 0.01) but monolinguals did not (*p* = 0.53)—and that monolinguals were more accurate than bilinguals for low NoS (*p* = 0.04), but not high NoS (*p* = 0.76). **Table 4** displays mean RT and accuracy values for monolinguals and bilinguals on the English task.

**Table 4:** Mean RT (SD) and accuracy (SD) for monolinguals and bilinguals in the lexical decision task in English and in the L1 (bilinguals).

		Monolinguals	Bilinguals (English)	Bilinguals (L1)
Reaction Time	High NoS	632.57 (*111.76*)	645.66 (*54.18*)	653.17 (*41.16*)
	Low NoS	668.82 (*122.58*)	659.98 (*61.59*)	657.76 (*54.83*)
Difference		-36.25^∗∗^	-14.33	-4.6
Accuracy	High NoS	98.32 (*2.27*)	98.57 (*2.45*)	98.57 (*1.77*)
	Low NoS	97.81 (*1.89*)	95.71 (*3.70*)	95.56 (*3.69*)
Difference		0.50	2.86^∗∗^	3.01^∗∗^


*^∗∗^*p* < 0.01.*

#### Monolinguals vs. Bilinguals: Performance in L1

Reaction time and accuracy were analyzed with a repeated measures ANOVA with the factors group (monolinguals vs. bilinguals) and condition (high vs. low), and the results revealed the same effects as the analysis of English performance. Specifically, a main effect of NoS was due to faster [*F*(1,33) = 14.33, *MSE* = 508.63, *p* < 0.01, $\eta_{p}^{2}$ = 0.30] and more accurate [*F*(1,33) = 8.21, *MSE* = 6.60, *p* < 0.01, $\eta_{p}^{2}$ = 0.20] responses in high NoS than low NoS items. A Group × NoS interaction for both RT [*F*(1,33) = 8.62, *MSE* = 508.63, *p* < 0.01, $\eta_{p}^{2}$ = 0.21] and accuracy [*F*(1,33) = 4.17, *MSE* = 6.60, *p* = 0.05, $\eta_{p}^{2}$ = 0.12], decomposed using Bonferroni *post hoc* analyses, was due to shorter RTs in high relative to low NoS items for monolinguals only, higher accuracy for high than low NoS items in bilinguals only, and higher accuracy in monolinguals relative to bilinguals only for low NoS items. **Table 4** reports the RT and accuracy values for the bilinguals in their L1.

#### Bilinguals: Performance in English vs. French

Reaction time and accuracy were analyzed with a repeated measures ANOVA with the factors Language (English, French) and NoS (high, low).

Reaction time analyses revealed a main effect of Language [*F*(1,17) = 13.31, *MSE* = 3773.79, *p* < 0.01, $\eta_{p}^{2}$ = 0.21], indicating faster reaction times in the English than in the French experiment. There was also a main effect of NoS [*F*(1,17) = 4.37, *MSE* = 1255.48, *p* = 0.05, $\eta_{p}^{2}$ = 0.21], indicating faster reaction times for high than low NoS words. The interaction between Language and NoS was not significant (*F* < 1).

Similarly, accuracy analyses revealed a main effect of Language [*F*(1,17) = 5.65, *MSE* = 20.54, *p* = 0.03, $\eta_{p}^{2}$ = 0.25], indicating greater accuracy in English than French. A main effect of NoS was also observed [*F*(1,17) = 18.09, *MSE* = 13.26, *p* < 0.01, $\eta_{p}^{2}$ = 0.53], indicating more accurate responses to high than low NoS words. The interaction between Language and NoS was not significant (*F* < 1). **Table 5** displays mean RT and accuracy for performance in English and French.

**Table 5:** Mean RT (SD) and accuracy (SD) for bilinguals in the lexical decision task in English and French, and in the L1 and L2.

		English	French	L1	L2
Reaction time	High NoS	645.66 (*54.18)*	695.35 (*78.31)*	653.18 (*41.16*)	687.83 (*89.70*)
	Low NoS	659.99 (*78.31)*	715.95 (*99.24*)	657.76 (*54.83*)	718.17 (*101.79*)
Difference		-14.33^∗^	-20.60	-4.58	-30.35^∗^
Accuracy	High NoS	98.57 (*2.45*)	96.83 (*2.16*)	98.57 (*1.77*)	96.83 (*2.75*)
	Low NoS	95.71 (*3.70*)	92.38 (*6.04*)	95.56 (*3.69*)	92.54 (*6.13*)
Difference		2.86^∗∗^	4.44^∗∗^	3.01^∗∗^	4.23^∗∗^


*^∗^*p* < 0.05; ^∗∗^*p* < 0.01.*

#### Bilinguals: Performance in L1 vs. L2

Overall, bilinguals responded more quickly [*F*(1,17) = 9.38, *MSE* = 4336.52, *p* < 0.01, $\eta_{p}^{2}$ = 0.36] and accurately [*F*(1,17) = 4.77, *MSE* = 21.33, *p* = 0.04, $\eta_{p}^{2}$ = 0.22] in their L1 than their L2. A main effect of NoS was due to faster [*F*(1,17) = 4.37, *MSE* = 1255.48, *p* = 0.05, $\eta_{p}^{2}$ = 0.21] and more accurate [*F*(1,17) = 18.09, *MSE* = 13.26, *p* < 0.01, $\eta_{p}^{2}$ = 0.52] responses to high than low NoS stimuli. In addition, with respect to RT, there was a trend toward an interaction between Language and NoS [*F*(1,17) = 4.23, *MSE* = 706.01, *p* = 0.055, $\eta_{p}^{2}$ = 0.20], demonstrating that responses were only faster for high than low NoS in the L2. **Table 5** displays mean RT and accuracy for bilinguals’ L1 and L2.

### Event-Related Potentials

Analyses were performed at electrode sites where the N400 is typically distributed topographically (midline centro-posterior sites) and included 50 ms time bins starting at 300 ms and ending at 600 ms post-stimulus onset. Additional analyses were also performed at left and right centro-posterior sites; however, results from these analyses are only reported if they provide additional information. Interactions were decomposed with Bonferroni adjusted simple effects comparisons and the Greenhouse–Geisser correction was used for analyses with more than 2 degrees of freedom in the denominator. As per convention, we report the unadjusted degrees of freedom, the adjusted *MSE* and the Greenhouse–Geisser epsilon value. In addition, given that we made specific hypotheses regarding the interaction between NoS and group/language, we examined the planned simple effects comparisons for the Group × NoS interaction, even when the interaction was not significant in the omnibus analysis.

#### Monolinguals vs. Bilinguals: Performance in English

**FIGURE 1:**
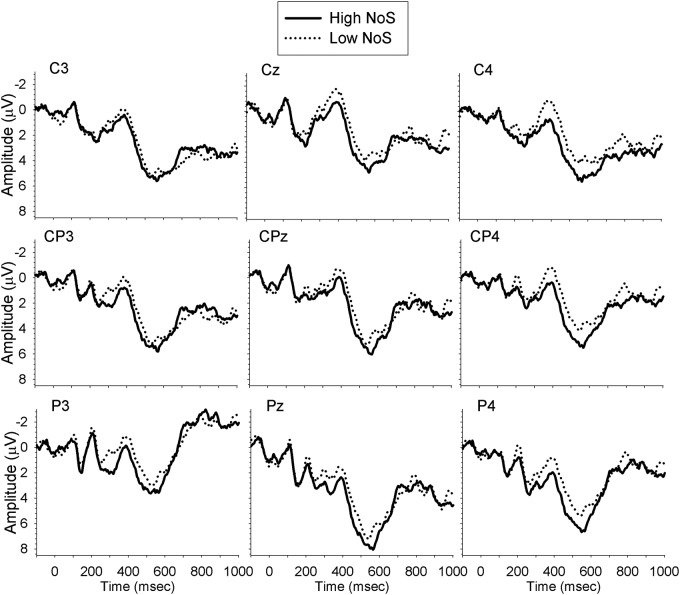
**Waveforms elicited by monolinguals performing the task in English**.

**FIGURE 2:**
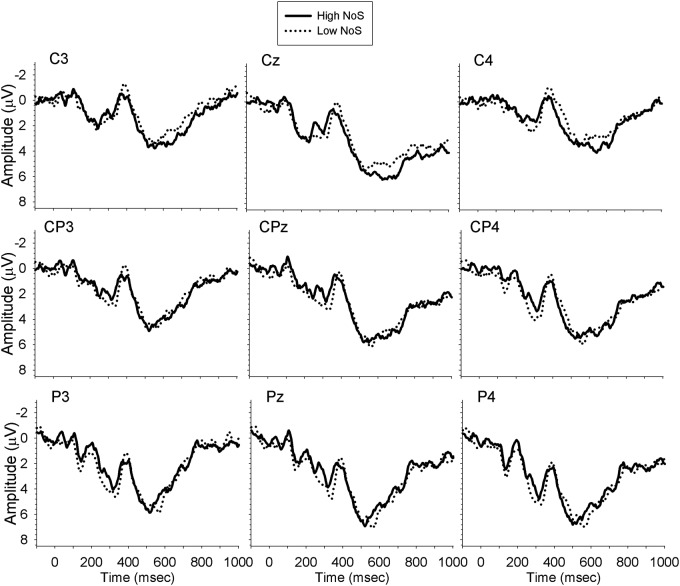
**Waveforms elicited by bilinguals performing the task in English**.

Analyses were carried out separately for the midline, and left and right lateral sites, with a mixed ANOVA with the within-subject factors Time (300–350, 350–400, 400–450, 450–500, 500–550, 550–600), Site (midline: Cz, CPz, Pz; left: C3, CP3, P3; right: C4, CP4, P4), NoS (high, low) and the between-subject factor Group (monolingual, bilingual). There were no significant effects of interest in the midline or left lateral electrode sites. However, analysis of the right lateral sites revealed a significant interaction between NoS and Time [*F*(5,165) = 2.93, *MSE* = 4.75, *p* = 0.03, $\eta_{p}^{2}$ = 0.08, ε = 0.74], demonstrating larger amplitude N400 for low NoS relative to high NoS words from 400–500 ms. The planned comparison of the Group × NoS interaction revealed that the difference in N400 amplitude between high and low NoS was significant for monolinguals (*p* = 0.04; mean amplitude difference = 1.15 μV), but not for bilinguals (*p* = 0.71; mean amplitude difference = 0.20 μV). **Figures 1** and **2** show the waveforms elicited by task performance in English in monolinguals and bilinguals, respectively.

#### Monolinguals vs. Bilinguals: Performance in L1

**FIGURE 3:**
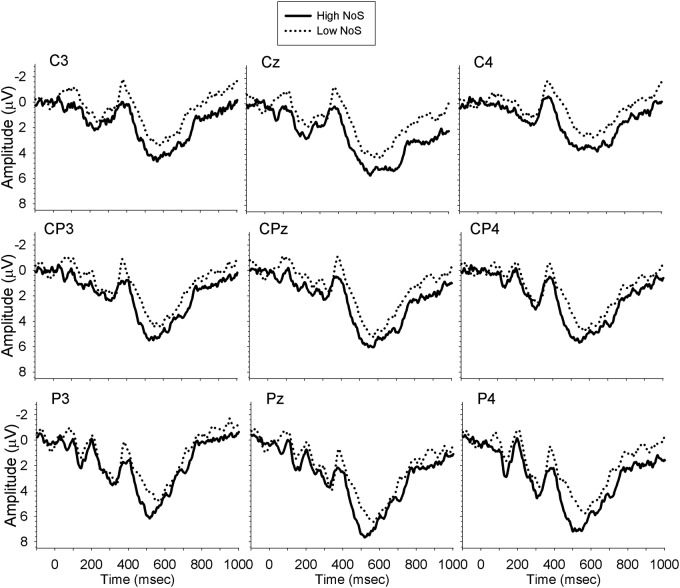
**Waveforms elicited by bilinguals performing the task in their L1**.

A mixed ANOVA with the factors of Group (monolinguals vs. bilinguals), Time (300–350, 350–400, 400–450, 450–500, 500–550, 550–600), Site (Cz, CPz, Pz), and NoS (high, low) revealed a main effect of condition whereby low NoS items elicited a larger N400 than high NoS items [*F*(1,33) = 4.20, *MSE* = 92.22, *p* = 0.05, $\eta_{p}^{2}$ = 0.11]. No other main effects or interactions were significant, and the analysis of the lateral sites did not provide any additional information. Planned comparisons of the Group × NoS interaction also did not yield any additional information. **Figure 3** shows the waveforms elicited by bilinguals’ performance in their L1.

#### Bilinguals: Performance in English vs. French

**FIGURE 4:**
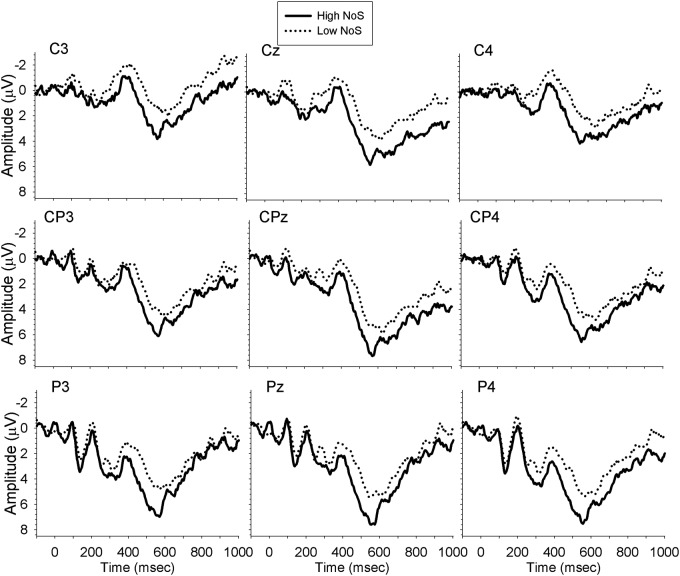
**Waveforms elicited by bilinguals performing the task in French**.

For midline sites, we computed a repeated measures ANOVA with the factors Time (300–350, 350–400, 400–450, 450–500, 500–550, 550–600), Site (Cz, CPz, Pz), NoS (high, low) and language (English, French). These analyses revealed a significant 3-way interaction of NoS, Time, and Site [*F*(10,170) = 2.61, *MSE* = 0.53, *p* = 0.04, $\eta_{p}^{2}$ = 0.13, ε = 0.42]. Simple effects comparisons revealed that from 450–500 ms, low NoS words elicited larger N400 amplitudes than high NoS words at all electrode sites, with the largest amplitude difference at site Pz. The planned comparison of the Language × NoS interaction showed that the French task elicited a larger amplitude N400 than the English task for low NoS words only, and that the effect of NoS was only significant in French. No additional information was obtained from the analysis of the lateral sites. **Figure 4** shows the waveforms elicited by bilinguals’ performance in French.

#### Bilinguals: Performance in L1 vs. L2

**FIGURE 5:**
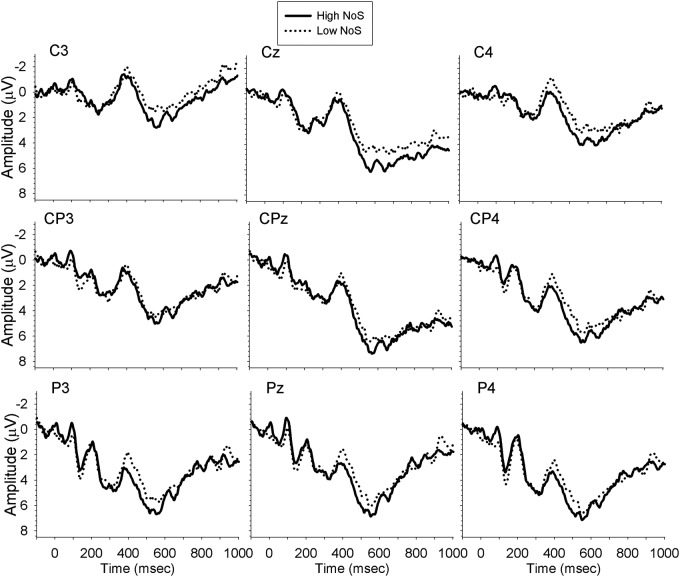
**Waveforms elicited by bilinguals performing the task in their L2**.

Data were analyzed using a mixed ANOVA with the factors of Time (300–350, 350–400, 400–450, 450–500, 500–550, 550–600), Site (Cz, CPz, Pz), NoS (high, low) and Language (L1 vs. L2). A trend for a main effect of NoS was observed in midline sites [*F*(1,17) = 4.07, *MSE* = 106.84, *p* = 0.06, $\eta_{p}^{2}$ = 0.19], whereby low NoS stimuli elicited larger N400s than high NoS items (note that this effect shows the same trend in both left (*p* = 0.06) and right (*p* = 0.06) sites). Planned simple effects comparisons of the Language × NoS interaction showed that the effect of NoS was only significant in L2 (*p* = 0.05). **Figure 5** shows the waveforms elicited by bilinguals’ performance in their L2.

For a summary of all results, see **Table 6**.

**Table 6:** Summary of behavioral and ERP results.

	RT	Accuracy	ERP/N400
Monolinguals vs.	NoS: high < low	NoS: high > low	NoS × Time: low > high
Bilinguals: English	Group: ns	Group: ns	Group × NoS (planned comparison): low > high for monolinguals only
	Group × NoS: high < low in monolinguals only (*p* = 0.06)	Group × NoS: high > low in bilinguals; monolinguals > bilinguals for low NoS	
Monolinguals vs.	NoS: high < low	NoS: high > low	NoS: low > high
Bilinguals: L1	Group: ns	Group: ns	Group × NoS (planned comparison): ns
	Group × NoS: high < low in monolinguals only	Group × NoS: high > low in bilinguals; monolinguals > bilinguals for low NoS	
Bilinguals: English vs. French	Language: English < French	Language: English > French	NoS x Time x Site: low > high 450-500 ms post-stimulus, largest at site Pz
	NoS: high < low	NoS: high > low	Group × NoS (planned comparison): French > English for low NoS; low > high in French only
	Language × NoS: ns	Language × NoS: ns	
Bilinguals: L1 vs. L2	Language: L1 < L2	Language: L1 > L2	NoS: low > high (*p* = 0.06)
	NoS: high < low	NoS: high > low	Group × NoS (planned comparison): low > high in L2 only
	Language × NoS: high < low in L2 only (*p* = 0.055)	Language × NoS: ns	


*ns, not significant; NoS, number of senses.*

## Discussion

The present study examined the effect of one measure of semantic richness—the number of senses (NoS) possessed by a word—on behavioral and ERP responses in English-speaking monolinguals and highly proficient bilingual speakers of English and French. Participants completed a lexical decision task with high- and low-NoS items in English (all participants) and French (bilingual participants only). We compared monolingual performance to bilingual performance first in English, and then in the first language (English for 10 participants and French for eight participants). Bilingual performance was then compared in the two languages (English vs. French and L1 vs. L2).

Overall, shorter RTs and higher accuracy were observed for high- than low-NoS items in the English task across all participants. Similarly, a larger amplitude N400 was revealed for low- relative to high-NoS items in right lateral sites; this difference was larger in monolinguals than bilinguals. When bilinguals’ L1 performance was compared to monolingual performance, shorter RTs and higher accuracy were again observed for high- relative to low-NoS items, and low-NoS items elicited greater N400 amplitude compared to high-NoS items. RT effects were stronger in monolinguals than bilinguals even when bilingual performance in the L1 was used in the analyses.

These findings indicate that both monolinguals and bilinguals exhibit semantic richness effects in a lexical decision task, as predicted; these effects are observed in both the English and the French tasks. However, the high-NoS advantage was more robust in monolinguals than bilinguals, as predicted: in the English-only analysis, larger differences in the ERP response were observed between conditions in monolinguals relative to bilinguals, and in the L1-only analysis, RT effects of NoS were stronger in the monolingual group. While accuracy effects were stronger in the bilingual group, this finding is likely due to ceiling effects in the monolingual group (accuracy exceeded 97% in both conditions in this group).

The results thus confirm our hypothesis that semantic richness effects would be observed in both monolinguals and bilinguals, but that they would be stronger in the former than the latter group. These findings are consistent with previous research indicating differences in semantic function in monolinguals and bilinguals in children (Cremer and Schoonen, 2013). Specifically, we argue that semantic representations may be more impoverished in bilinguals relative to monolinguals, possibly due to less language experience in each of their languages. This account is consistent with the frequency lag hypothesis (Gollan et al., 2008), which holds that bilingual disadvantages in language processing result from lower experience with words in one language relative to monolinguals. Because any given word form thus has lower relative frequency for bilinguals than monolinguals, lexical retrieval is slower and less accurate. We propose that semantic representations are also less elaborated in bilinguals than monolinguals as a result of this reduced language experience. Specifically in the present case, bilinguals may have been exposed to fewer senses of a polysemous lexical item, leading to reduced effects of number of senses.

We also examined bilinguals’ performance in each of their languages. Behaviorally, bilinguals showed shorter RTs and higher accuracy in English than French. As predicted, high NoS words were recognized more quickly and accurately than low NoS words. N400 effects were also consistent: low NoS words elicited larger N400 amplitudes than high NoS words. Interestingly, this effect was stronger in French than English even though behavioral performance was superior in English. In order to tease apart the language effects, we then analyzed bilinguals’ performance in their first and second languages. Overall, bilinguals’ behavioral performance was stronger in the first than the second language, and the predicted NoS effects were observed overall in both behavioral and ERP measures, albeit slightly more strongly in the second language.

These results suggest a complex picture of language dominance in this highly proficient bilingual sample, with stronger behavioral performance in English in the context of stronger ERP effects in French. Assessing language dominance is complex because language proficiency is multifactorial (Sheng et al., 2014); the present findings suggest that neural and behavioral performance on a semantic task may reveal different patterns of dominance. We suggest that the N400 NoS effect, which was stronger in French than English, reflects superior semantic knowledge in French, while superior behavioral performance in English may reflect stronger lexical- or decisional-level performance in English. It is particularly interesting that NoS effects are stronger overall in the second language; this finding indicates that the native language is not necessarily the language in which semantic representations are most elaborated. It is of course possible that the differing performance in English and French may be due to differences in the experiments themselves. For example, the overall degree of semantic richness may differ between English and French. This confound is unavoidable, because stimulus lists must differ across the two languages. However, the fact that neural and behavioral responses were dissociated in the two languages suggests that the differing performance is not due purely to differences in test materials. Future research should examine these effects in other language pairs and in speakers of differing degrees of dominance in order to assess the generalizability of the current findings to other bilingual populations.

In sum, as predicted, we found effects of NoS across both participant groups, and these effects were stronger in monolinguals than in bilinguals. This finding suggests that semantic representations are more elaborated in monolinguals than bilinguals, consistent with previous research on frequency effects (Gollan et al., 2008). Moreover, we found dissociations across languages in bilinguals, with stronger behavioral NoS effects in English and stronger ERP NoS effects in French. This finding points to the dissociation in different processing stages in the two languages, and suggests that different aspects of linguistic performance may be stronger in each of a bilingual’s two languages.

## Author Contributions

VT: Intellectual conceptualization, analyses, and manuscript drafting. RL: Intellectual contribution, data collection, analyses, and manuscript drafting. SK: Intellectual contribution, analyses, and manuscript drafting.

## Conflict of Interest Statement

The authors declare that the research was conducted in the absence of any commercial or financial relationships that could be construed as a potential conflict of interest.

## Footnotes

1 Monolingual participants’ performance was reported in Taler et al. (2013).
